# Is It Time to Start Transitioning From 2D to 3D Cell Culture?

**DOI:** 10.3389/fmolb.2020.00033

**Published:** 2020-03-06

**Authors:** Caleb Jensen, Yong Teng

**Affiliations:** ^1^Department of Oral Biology and Diagnostic Sciences, Dental College of Georgia, Augusta University, Augusta, GA, United States; ^2^Department of Biology, College of Science and Mathematics, Augusta University, Augusta, GA, United States; ^3^Georgia Cancer Center, Medical College of Georgia, Augusta University, Augusta, GA, United States; ^4^Department of Medical Laboratory, Imaging and Radiologic Sciences, College of Allied Health, Augusta University, Augusta, GA, United States; ^5^Department of Biochemistry and Molecular Biology, Medical College of Georgia, Augusta University, Augusta, GA, United States

**Keywords:** 3D cell culture, biomedical and drug research, advance and progress, methods and applications, techniques

## Abstract

Cell culture is an important and necessary process in drug discovery, cancer research, as well as stem cell study. Most cells are currently cultured using two-dimensional (2D) methods but new and improved methods that implement three-dimensional (3D) cell culturing techniques suggest compelling evidence that much more advanced experiments can be performed yielding valuable insights. When performing 3D cell culture experiments, the cell environment can be manipulated to mimic that of a cell *in vivo* and provide more accurate data about cell-to-cell interactions, tumor characteristics, drug discovery, metabolic profiling, stem cell research, and other types of diseases. Scaffold based techniques such as hydrogel-based support, polymeric hard material-based support, hydrophilic glass fiber, and organoids are employed, and each provide their own advantages and applications. Likewise, there are also scaffold free techniques used such as hanging drop microplates, magnetic levitation, and spheroid microplates with ultra-low attachment coating. 3D cell culture has the potential to provide alternative ways to study organ behavior via the use of organoids and is expected to eventually bridge the gap between 2D cell culture and animal models. The present review compares 2D cell culture to 3D cell culture, provides the details surrounding the different 3D culture techniques, as well as focuses on the present and future applications of 3D cell culture.

## Background

**FIGURE 1:**
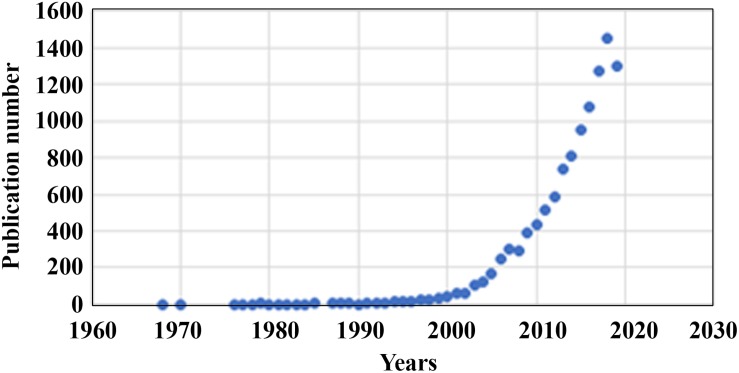
Number of publications per year (1968–2020) on 3D cell cultures gathered from PubMed.

Two dimensional (2D) cell culture has been the method used to culture cells since the early 1900s (Ferreira et al., 2018), which plays a vital role in research but has many limitations due to 2D models inaccurately representing tissue cells *in vitro* (Costa et al., 2016). Another method known as 3D cell culture has shown improvements in studies targeted toward morphology, cell number monitoring, proliferation, response to stimuli, differentiation, drug metabolism, and protein synthesis (Antoni et al., 2015). All of this is made possible by 3D cultures’ capability to model a cell *in vivo* while being cultured *in vitro* (Ravi et al., 2015). 3D cell culture has many applications such as cancer research, stem cell research, drug discovery, and research pertaining to other types of diseases, which is more popular today than ever (Figure 1). Table 1 compares the different aspects of 2D and 3D cell culture and explains the advantages and disadvantages of both methods. Furthermore, 3D culture offers several methods of cell culture depending on the type of experiment being performed.

**TABLE 1:** Comparison of 2D and 3D cell culture.

**Important characteristics**	**2D cell culture**	**3D cell culture**	**References**
Cell shape	• Cells shape is flat and elongated since the cells can only grow and expand two dimensionally • Cells grow into a monolayer on the plate	• Natural cell shape is preserved and cell growth • Cells grow into 3D aggregates/spheroids • Spheroids contain multiple layers	Costa et al., 2016; Langhans, 2018
Cell exposure to medium	• All cells in the culture receive the same amount of nutrients and growth factors from the medium in the plate • This causes more cells to be in the same stage of the cell cycle	• Nutrients does not have to be equally divided amongst all cells but can be if needed • The core cells often remain inactive since they receive less oxygen and growth factors from the medium • This process resembles the core cells in tumor cells, making it possible to mimic the behavior and structure of a tumor cell *in vivo*	Dhaliwal, 2012; Costa et al., 2016; Langhans, 2018
Cell junction	• Cell junctions are less common and less accurately represent real junctions	• Cell junctions are common and allow for cell-to-cell communication • Cells communicate through exchange ions, small molecules, and electrical currents	Pontes Soares et al., 2012; Ravi et al., 2015; Costa et al., 2016; Langhans, 2018; Lang et al., 2019
Cell differentiation	• Cell differentiation is poor	• Cells are well differentiated	Imamura et al., 2015; Costa et al., 2016; Langhans, 2018
Drug sensitivity	• Cells often have little resistance to drugs making it appear as though drugs administered to the cells were a successful treatment • Drugs are not well metabolized	• Cells often have more resistance to drug treatment • Drug metabolism is much better • Gives a more accurate representation of the drug’s effects	Haisler et al., 2015; Imamura et al., 2015; Langhans, 2018
Cell proliferation	• Cells proliferate at an unnaturally rapid pace.	• Proliferation rates are realistic and can be high or low depending on technique and types of cells being studied.	Ravi et al., 2015, Langhans, 2018
Expression levels	• Gene and protein expression levels are often vastly different compared to *in vivo* models	• Gene and protein expression levels resemble levels found from cells *in vivo*	Ravi et al., 2015; Costa et al., 2016; Langhans, 2018
Cost	• For large-scale studies, it is much cheaper than using 3D culture	• Are typically more expensive than 2D cell culture techniques and require more time • 3D cell culturing reduces the differences between *in vitro* and *in vivo* drug screening, decreasing the likelihood of needing to use animal models	Ravi et al., 2015; Costa et al., 2016; Langhans, 2018
Apoptosis	• Drugs can easily induce apoptosis in cells	• Higher rates of resistance for drug-induced apoptosis	Costa et al., 2016
Response to stimuli	• Inaccurate representation of response to mechanical stimuli of cells • Cells cannot experience gravity since they are unable to expand into the third dimension	• Accurate representation of response to mechanical stimuli of cells • Cells can experience gravity giving a more accurate representation of a cell *in vivo*	Ravi et al., 2015; Costa et al., 2016
Usage and analysis	• Highly replicable and easily interpretable • Better for long-term cultures	• Can be difficult to replicate experiments • Can be difficult to interpret data	Kapałczyńska et al., 2018

**FIGURE 2:**
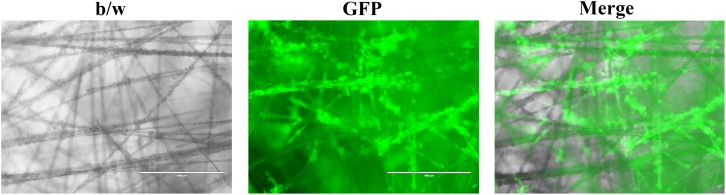
Representative images of cancer cells growing in the SeedEZ scaffold, a new 3D culture system with transparent glass microfibers. Head and neck cancer HN17 cells expressing green fluorescent protein (GFP) were seeded in SeedEZ for 7 days, and images were taken under a fluorescence microscope.

Scaffold based techniques such as hydrogel-based support, polymeric hard material-based support, hydrophilic glass fiber, and organoids provide an array of advantages. Hydrogels are unique because of their ability to mimic the ECM while allowing soluble factors such as cytokines and growth factors to travel through the tissue-like gel (Langhans, 2018). Hydrogels are also versatile since they can be used to create spheroids and can be prepared in multiple ways depending on the experiment being performed. Both natural and synthetic hydrogels exist, with natural gels commonly being made with natural polymers such as fibrinogen, hyaluronic acid, collagen, Matrigel, gelatin, chitosan, and aginate (Dhaliwal, 2012). Natural gels made of collagen have been used to model 3D tumors via MCTS where the cells were embedded in the gel (Van-Minh et al., 2016). The study concluded that the 3D model allowed for drug screening as well as noticed differences in cell shape, density, and drug sensitivity when compared to cells cultured on the traditional monolayer (Van-Minh et al., 2016). Synthetic hydrogels are typically made with synthetic polymers made from polyethylene glycol (PEG), polylactic acid (PLA), or poly(vinyl acetate) (PVA) (Dhaliwal, 2012). Polymeric hard scaffolds are an important tool in studying cell-to-ECM interactions due to the scaffold’s ability to replicate the structure of the ECM. A study showed that HepG2 liver cells cultured using a 3D polymeric hard scaffold were less affected by cytotoxic compounds and had greater viability than those grown in 2D. Furthermore, polymeric hard scaffolds are extremely useful in studying tissue regeneration as well as testing tumor cell treatments. Hydrophilic glass fibers are important for modeling 3D tumors testing antibodies, invasion, as well as tracking cell migration. SeedEZ discovered by Lena Biosciences is such an inert and transparent glass microfiber scaffold (Figure 2), which allows for various cell types to be seeded at once in order to create different 3D layers within the cell. Compared with other 3D cell culture systems, such as 3D Matrigel culture drops, SeedEZ promotes cell-cell interaction and formation of 3D cell network more efficiently. By taking these advantages, SeedEZ represents the most effective tools for cancer research and drug testing (Lang et al., 2019). The use of hydrophilic glass fibers are still to be further explored, but offer an abundance of potential. Organoids aggregate into spheroids by forming ECM fibers that link single cells together via integrin binding and mimic the microenvironment of certain organs to allow researchers to model human diseases through the use of patient-derived pluripotent stem cells (Yin et al., 2016). Furthermore, researchers are able to grow tumor models using organoids through the use of patient derived tissue cancer cells. This allows scientists to model the patient’s tumor in order to test treatments on a patient-to-patient basis. Lastly, organoids have shown signs that one day they may be able to aid in an alternative organ transplantation method. Organoids are changing the way in which researchers study human development, as well as test new disease treatments.

Scaffold-free techniques including hanging drop microplates, magnetic levitation, and spheroid microplates with ultra-low attachment coating are unique in their ability to freely grow with no scaffold and provide special advantages as a result. Hanging drop plates allow the formation of spheroids via self-aggregation through the use of gravity. The spheroids hang in open bottomless wells which are often enclosed in the bottom of the plate in order to regulate the environmental humidity of the cells. Hanging drop plate methods have a wide range of uses due to their replicability. A study was conducted in which cardiac spheroids were created by co-culturing endothelial cells, fibroblasts, and cardiomyocytes derived from induced pluripotent stem cells (Langhans, 2018). The results showed a cell culture model in which toxic effects in human heart tissue could be studied due to how closely the cardiac spheroids resembled *in vivo* features of the human heart (Langhans, 2018). Magnetic levitation is performed by injecting cells with magnetic nanoparticles allowing cells aggregate into a spheroid when exposed to an external magnet. This creates a concentrated cell environment in which ECM can be synthesized, and analyzation via western blotting and other biochemical assays can be performed (Haisler et al., 2015). Furthermore, the external magnet can be used manipulate the 3D culture, allowing for special control and more advanced environments. Overall, magnetic levitation allows both basic and advanced environments to be replicated, thus making it a very versatile technique (Haisler et al., 2015). Spheroid microplates with ultra-low attachment coating are commonly used to study tumor cells as well as grow multicellular cultures due to the large volume (Imamura et al., 2015). Studies show that multicellular spheres that were grown from two NSCLC cells display very different growth characteristics when compared to 2D cell cultures. The cells exhibited multidrug resistance, displayed stem-cell like traits, and cell motility was increased (Imamura et al., 2015). Furthermore, tumor cells derived from breast cancer cells display *in vivo* characteristics that are useful when testing treatments (Imamura et al., 2015).

A common tool used in research is the use of animal models. Mouse models are commonly used in research to test new drugs and treatment strategies especially in cancer research. 3D culturing techniques have allowed researchers to model tumors and organs in order to perform drug treatment tests on them. Experts suggest that as these models continue to improve and become more commonplace, less animal models will need to be used.

3D cell culturing methods are beginning to outperform old 2D cell culture methods despite the fact that 3D culture is still in its infancy stages. Furthermore, each 3D culturing method comes with a unique set of advantages that can be implemented depending on the desired experiment. Table 2 displays a comparison between hydrogel-based support, polymeric hard material based support, hydrophilic glass fibers, magnetic levitation, and spheroids with ultra-low attachment coatings.

**TABLE 2:** Advanced 3D cell culturing technique comparison.

	**Function**	**Preparation**	**Advantages and Applications**
Hydrogel-based support	1. ECM can be replicated (Antoni et al., 2015) 2. Can be loaded with biological fluids and water (Antoni et al., 2015) 3. Can osmoregulate (Godugu et al., 2013)	1. Chemical crosslinking, free radical polymerization, irradiation crosslinking, and physical crosslinking via polyelectric complexation, hydrogen bonding, and hydrophobic association (Godugu et al., 2013)	1. Smart hydrogels can respond to environmental stimuli such as changes in temperature, pH, ionic strength, radiation, metal, electric field and more (Godugu et al., 2013) 2. Intestinal flow and diffusive transport (Langhans, 2018) 3. Act as drug storehouses, tissue barriers, and a bioactive moieties delivery system that stimulates the natural reparative process (Torkian et al., 2004; El-Sherbiny and Yacoub, 2013)
Polymeric hard material based support	1. The scaffold is used to replicate the *in vivo* ECM since cells can attach and form 3D cultures (Dhandayuthapani et al., 2011)	1. The cells are matured on the scaffold to model tumors or tissue (Shantha and Harding, 2003) 2. The cells are then cut to a diameter that fits inside a given test vessel (Hoffman, 2001)	1. The cell treatment procedures are very similar to 2D cell culture (Hoffman, 2001) 2. Very reproducible (Costa et al., 2016) 3. Tumoroids grown using patient samples show promising signs for drug screening and drug development (Peppas et al., 2000) 4. Tissue regeneration in bone, ligaments, cartilage, skeletal and vascular muscle, and central nervous system tissue (Haycock, 2011)
Hydrophilic glass fiber	1. Model the ECM (Cushing and Anseth, 2007) 2. Can be used in migration, invasion, chemo-invasion, and angiogenesis assays (Cushing and Anseth, 2007)	1. Commonly performed using the SeedEZTM lab device by Lena Biosciences 2. 3D cell cultures will be more consistent in shape, spread, thickness, and cell distribution in the X, Y, and Z dimensions (Cushing and Anseth, 2007)	1. Can perform spot culture experiments, mixed cell cultures, sol-state gel suspension experiments, non-contact and contact co-culture methods via the three-dimensional feeder layer technique, stack and culture experiments, and side-by-side cultures (Cushing and Anseth, 2007) 2. Cells may be primary cells, secondary cells, and cell lines of various origins and sources (Cushing and Anseth, 2007) 3. Can culture advanced 3D tumor models for long durations of time *in vitro* (Cushing and Anseth, 2007)
Magnetic levitation	1. The magnetic forces allow cell aggregation while inducing ECM synthesis (Godugu et al., 2013) 2. Promotes cell-cell interaction (Godugu et al., 2013)	1. Created by loading the cells with magnetic nanoparticles and then are exposed to an external magnetic field that causes cells to aggregate into a spheroid (Souza et al., 2010; Talukdar and Kundu, 2012)	1. Does not require a specific medium (Talukdar and Kundu, 2012) 2. Works with normal 2D cell culture techniques (Talukdar and Kundu, 2012) 3. Works with a wide range of cell types (Souza et al., 2010) 4. Not just limited to 96 well-plates (Adine et al., 2018) 5. Takes about 16 h for spheroids to form (Talukdar and Kundu, 2012) 6. Can form a 3D culture without the use of an artificial protein substrate (Talukdar and Kundu, 2012) 7. Can synthesize ECM while forming (Talukdar and Kundu, 2012)
Spheroid microplates with ultra-low attachment coating	1. The ultra-low attachment coating reduces cell adherence to promote spheroid formation (Dhandayuthapani et al., 2011) .	1. Typically made out of polystyrene and treated with hydrophilic or hydrophobic coatings or made with natural polymers such as agarose (Haisler et al., 2015) 2. The v-shaped bottomed wells promote consistent spheroid formation in all the wells (Dhandayuthapani et al., 2011)	1. Transfer of spheroids to a new plate is often unnecessary due to the large volume 96- or 384-well plates (Haisler et al., 2015; Imamura et al., 2015) 2. The spheroids of human breast cancer cells mimicked characteristics *in vivo* such as hypoxia, dormancy, anti-apoptotic features, and drug resistance in one study (Coleman et al., 2007) 3. 3D neurospheres have proven useful in studying growth kinetics and drug toxicity (Imamura et al., 2015)

## 3D Cell Culture for Drug Discovery

Drug discovery is the most important aspect in the fields of medicine and pharmacology, but often takes an extensive amount of time as well as money and yields low success rates when testing new medicines in animal models and in preclinical trials (Langhans, 2018). Due to low success rates, less than half of all drugs in Phase II and Phase III clinical trials are successful indicating the desperate need for new methods and technologies that improve the efficacy of drug discovery (Langhans, 2018). Animal models tend to be expensive, whereas assays using cultured cells have proven to be to be easily replicable, quick, and cost-effective (Costa et al., 2016). The most commonly used method in drug discovery to date is the use of 2D cell cultures (Costa et al., 2016). 2D cell cultures have aided in the discovery of many biological and disease processes but are unable to mimic the complicated microenvironment cells experience in tissue (Costa et al., 2016; Lv et al., 2017). Drug discovery relies on understanding the link between cells and the ECM in which they interact (Cushing and Anseth, 2007). ECM molecules include matrix proteins, glycoproteins, glycosaminoglycans, proteoglycans, ECM-sequestered growth factors, vascular endothelial growth factor (VEGF), platelet-derived growth factor (PDGF), hepatocyte growth factor (HGF) as well as other secreted proteins (Breslin and O’Driscoll, 2013). These growth factors and proteins play key roles in regulating cell proliferation, migration, differentiation, adhesion, and survival (Bonnans et al., 2014). Furthermore, the structure of the ECM can affect the cell’s response to drugs by changing a drug’s mechanism of action, amplifying drug efficacy, or by boosting the cells affinity for drug resistance (Bonnans et al., 2014).

In order to predict the effectiveness of a drug on a cell, a 3D culture model would have to mimic the microenvironment of tissue in which cells can proliferate, aggregate, and differentiate (Lv et al., 2017). Cells cultured in 3D have displayed different responses to drugs than cells cultured in 2D for several reasons. Differences in physical and physiological properties between 2D and 3D cultures cause 2D cells to be more susceptible to the effects of drugs than 3D cells due to the fact that 2D cells are unable to maintain a normal morphology like 3D cells can (Cushing and Anseth, 2007; Lv et al., 2017; Langhans, 2018). Another reason 2D cells are more sensitive to drugs than 3D cells is because of the difference in the organization of surface receptors on the cell (Cushing and Anseth, 2007; Bonnans et al., 2014; Lv et al., 2017; Langhans, 2018). Drugs often target certain receptors on the surface of cells (Langhans, 2018). Differences in structure and spacial arrangement of surface receptors likely effect the binding efficacy of drugs to the receptors eliciting different responses (Lancaster and Knoblich, 2014; Langhans, 2018). Third, cells cultured in 2D are often all at the same cell stage whereas 3D cells are often in different cell stages much like cells *in vivo* (Langhans, 2018). In 3D cells, difference in cell stage likely means that there are proliferating cells available in the outer region in the cell (Bonnans et al., 2014). Many drugs require cell proliferation to be effective, favoring 3D cell culture (Langhans, 2018). Lastly, the difference in shape between 2D and 3D cells causes a difference in local pH levels within the cells as a result of 3D cells having greater depth than 2D cells (Lancaster and Knoblich, 2014). It has been proven that lower intracellular pH levels cause a reduction in drug efficacy, contributing to drug resistance (Lancaster and Knoblich, 2014).

Metabolic profiling is used to demonstrate metabolic cooperation between varying cell types and is becoming a popular technique in 3D culture models due to the accuracy of the results when compared to cells *in vivo* (Tung et al., 2011). Previously, 2D culture models have been used to test cancer metabolism but recent studies suggest 3D culture models provide more insight when testing the efficacy of new drugs (Russell et al., 2017). Through profiling, researchers have discovered that drug treatments sometimes kill all the cells in 2D culture monolayer but only kill some of cells that make-up the protective layer of spheroids in 3D models (Russell et al., 2017). The extra dimension in 3D culture has helped researchers understand the flaws present in 2D models that cause lower rates of drug efficacy relative to *in vivo* trials (Ferrick et al., 2008). Furthermore, researchers have concluded that metabolic profiling in 3D culture is inherently different than 2D culture due to a reduced sensitivity to ATP synthase (a common metabolic inhibitor) (Ferrick et al., 2008). Researchers concluded that this difference would cause a distinction in metabolic profiles due to differences between the responses to various chemotherapeutics in 2D and 3D (Tung et al., 2011).

3D cell culture has become one of the top methods of choice in drug discovery due to the fact that 3D cell cultures allow cell-to-cell and cell-to-matrix interaction much like the interactions cells *in vivo* experience. Furthermore, 3D culture models using human cells avoids the use of mouse models which are often expensive and inaccurately depict the effectiveness and side effects of drugs (Langhans, 2018).

## Utilization of Stem Cells to Fabricate 3D Spheroids and Organoids

Stem cells are commonly used as a means of regenerative medicine and cell therapy applications. In clinical applications however, 2D cell culture techniques have proven to be ineffective when using stem cells (Lv et al., 2017). This is a result of the 2D culture’s inability to accurately replicate the *in vivo* microenvironment of stem cells. Furthermore, MSCs often decrease in replicative ability as time progresses when culturing in 2D, invalidating any chance of them being used in large scale randomized clinical trials despite them showing beneficial effects in small-scale studies (Cushing and Anseth, 2007). When cultured in spheroids however, MSCs display a different morphology than 2D cultured MSCs (Cushing and Anseth, 2007). MSCs cultured in spheroids have gene expression patterns unlike those cultured in 2D. They display the upregulation of multiple genes associated with stress response, inflammation, redox signaling, hypoxia, and angiogenesis (Potapova et al., 2007).

Through the use of spheroid cultures, MSC-based therapeutics have greatly improved. In relation to 2D cultures, MSC spheroid cultures showed improvements such as an increase in the paracrine secretion of cytokines, more robust antiapoptotic and antioxidative capacities, and rising levels of ECM proteins (Cushing and Anseth, 2007). In addition, anti-inflammatory, tissue regenerative and reparative effects, and higher rates of posttransplant survival of MSCs have been observed as a result of MSC spheroid cultures (Potapova et al., 2007). To further test the effectiveness of MSC spheroids, MSC spheroids were injected into the kidneys of mole rats with ischemia reperfusion-induced acute kidney injury where the results were recorded (Cesarz and Tamama, 2016). The results post-injection showed that compared to 2D cultured cells, MSC spheroids were more effective in guarding the kidney against apoptosis, lessening tissue damage, bolstering vascularization, and alleviating renal function compared with 2D cultured cells (Cesarz and Tamama, 2016).

It has been recently discovered that pluripotent stem cells can be used to grow organoids that could potentially be used as a source of analogous tissue for transplantation in humans someday (Fordham et al., 2013). Researchers have successfully demonstrated that renal organoids derived from pluripotent stem cells can be transplanted under the renal capsules of adult mice (Cushing and Anseth, 2007; Lee C.T. et al., 2017). The organoid resembled the structures of a kidney *in vivo* and upon transplantation, the glomeruli were vascularized quickly showing promising signs toward an alternative kidney replacement strategy (Cushing and Anseth, 2007; Fordham et al., 2013). Using organoids derived from pluripotent stem cells, the future for alternative organ transplants in other organs is still being researched but remains optimistic.

Organoids play an increasingly important role in the study of genetic diseases due to their ability to model different regions of the body. For example, a rectal organoid was used to model cystic fibrosis to study the effects of the transmembrane conductance regulator-modulating compounds and another set of tubular organoids was used to model kidney disease where it was found that the microenvironment played a key role in the cyst formation (Cruz et al., 2017). Furthermore, organoids have proven to be useful models when studying neurodegenerative diseases such as Alzheimer and Parkinson disease (Dhaliwal, 2012; Cruz et al., 2017). Brain organoids generated from pluripotent stem cells taken from Alzheimer patients when treated with β- and γ-secretase inhibitors, showed promising therapeutic effects (Dhaliwal, 2012).

Lee Rubin, Ph.D. from the University of Harvard has begun to mass produce brain spheroids, as well as derive spheroids from patient cells to create a biobank in which treatments can be tested on a patient-to-patient basis (Rigamonti et al., 2016). By growing an unlimited supply of tissue, rare diseases can be tested on endlessly in an effort to find cures. Paola Arlotta, Ph.D. is another researcher from Harvard who allows organoids to grow for long periods of time so that they can develop into multi-thousand brain cell organoids that contain several brain cell types (Quadrato et al., 2017). This has allowed her to extensively study brain cell interactions with each other, to help understand how the cells communicate (Quadrato et al., 2017). Furthermore, researchers have found a link between autism and irregularities in the regulation of genes that play a role in proliferation using organoids derived from patients with autism (Forsberg et al., 2018). Although no cure has been found for autism, 3D cell culture techniques such as organoids have allowed researchers to take the first step in the direction of improvement and will continue to help uncover the mysteries behind many of the diseases people face.

## Advancement in Real-Time Visualization Via Microfluidic Systems Inspire Organ on a Chip Model

Real time visualization and analysis can play an important role in many different types of experiments. An experiment was conducted in which a 3D microfluidic system was created to mimic the microenvironment of a cell during angiogenesis via the use of a hydrogel scaffold (Vickerman et al., 2008). The hydrogel was microinjected into the microfluidic system allowing for control over surface shear stress, the flow of interstitial fluid through the matrix, the effects of the cell culture scaffold, gradients involving non-reactive solutes, as well as allowed cells to be monitored in real time (Vickerman et al., 2008). Three different extracellular capillary morphogenesis assays were performed, and time-lapse videos were taken of the cells in real-time to provide evidence of the multifunctionality of the 3D microfluidic device (Vickerman et al., 2008). The implementation of the hydrogel allowed the cells to be cultured within the microfluidic device giving the user more control over the microenvironment.

Perfusion systems have become a common way to replicate and monitor *in vivo* environments. In one investigation, a perfusion 3D cell culture microfluidic chip was created to monitor, and record real-time impedimetric biosensor changes as a result of cellular responses in oral cancer cells (Lei et al., 2014). Using a 3D agarose scaffold, the cells were encapsulated and cultured in a small chamber under perfusion of culture medium (Lei et al., 2014). The microenvironment was effective for studying cell proliferation and chemosensitivity of anti-cancer drugs in a non-invasive and real-time manner (Lei et al., 2014).

Recent advancements of microfluidic chips have led to what is known as the organ-on-a-chip model (also known as organ chips) (Sontheimer-Phelps et al., 2019). These organ chips overcome many difficulties currently presented in spheroids and organoids grown in ECM gels (Sontheimer-Phelps et al., 2019). Although spheroids and organoids are useful ways to model many types of cancers, they present limitations due to the lack of tissue-tissue interfaces and organ-level structures (Sontheimer-Phelps et al., 2019). Organ chips are created using computer microchip fabrication and are populated with living cells that resemble *in vivo* organ-level physiology and pathophysiology. This is made possible by constructing tissue-level and organ-level structures *in vitro* that function like tissues and organs do *in vivo* (Sontheimer-Phelps et al., 2019). Not only do organ chip models allow for better organ models, but they also permit high-resolution and real-time imaging making it easier to analyze *in vitro* biochemical, genetic, and metabolic activities present in human tissue (Sontheimer-Phelps et al., 2019). Some human organs that have been successfully modeled on organ chip devices include: kidney tubules (Maschmeyer et al., 2015), small intestine (Kasendra et al., 2018), bronchioles (Benam et al., 2016), liver (Beckwitt et al., 2018), BBB (Adriani et al., 2017), lung alveoli (Stucki et al., 2015), and bone marrow (Sieber et al., 2018). Not only can these organs be modeled, but more importantly they can give accurate organ-level responses to many stimuli including drugs (Hassell et al., 2017), toxins (66), radiation (Jalili-Firoozinezhad et al., 2018), cigarette smoke (Benam et al., 2016), and pathogens (Kim et al., 2016). Furthermore, therapeutic strategies and drug development are being tested on organ-chip models that mimic organs with diseases such as thrombosis (Barrile et al., 2018), inflammatory bowel disease (Kim et al., 2016), asthma (Benam et al., 2016), and barth syndrome (Wang et al., 2014).

A highly detailed experiment was conducted that constructed a blood-brain barrier chip (BBBC) model that mimicked the *in vivo* structure of micro blood vessels in the brain through the use of a type 1 collagen hydrogel (Yu et al., 2019). Endothelial cells, pericytes, and astrocytes from neonatal rats were cocultured in the collagen matrix to study cell interactions in the brain microvasculature as well as test new drugs for neurovascular diseases (Yu et al., 2019). The BBBC fluid flow used gravity and resistance in a paper-based resistor as a driving force rather than a pump (Yu et al., 2019). The fluid flow made the BBB more accurate than previous static 2D models since the media flow provided mechanical cues and facilitated mass transfer allowing functional maintenance of the primary endothelial cells (Yu et al., 2019). The BBBC also allowed for immunofluorescence imaging which helped confirm the formation and accuracy of the BBB (Yu et al., 2019). The results yielded that this BBBC model was effective for *in vitro* functional studies as drug screening for drugs that target or protect the BBB (Yu et al., 2019).

Another idea that has gained immense popularity amongst organ chip researchers is the idea of a human-on-a-chip (Wang et al., 2020). This model aims to examine normal human physiology within a microfluidic system by combining single organ chip designs into a multi-organ chip design that allows the organs to work in conjunction with each other much like organs in the human body do (Wang et al., 2020). MOC and a complete human-on-a-chip design would allow for cheaper and more effective drug testing and thus would greatly benefit biomedical sectors (Wang et al., 2020).

A revolutionary technique was carried-out that successfully manufactured a lung/liver-on-a-chip by connecting liver spheroid cultures with a 3D organotypic bronchial model (Vickerman et al., 2008). The experiment aimed to study the effect of certain aerosols on the lungs and incorporated the liver spheroid model to test the potential toxicity of the aerosols as well as their metabolites (Vickerman et al., 2008). The liver model was built using human HepaRG cells and the lung model was constructed with normal human bronchial epithelial cells (Vickerman et al., 2008). The study concluded that this MOC model was effective for demonstrating the assessment of compound toxicity on both the lungs and liver, and that it was relatively easy to use and maintain (Vickerman et al., 2008).

Another experiment successfully fashioned a MOC that connected models of the GI tract and liver through the use of 3D cell culture (Lei et al., 2014). The 3D liver model was created by using a polymer scaffold in which a human hepatocellular carcinoma cell line (HepG2 C3A) was cultured on (Lei et al., 2014). The primary hIECs were derived from patients during a colonoscopy screening and cultured in a 3D matrigel culture (Lei et al., 2014). The GI and liver compartments of the chip were connected via gravity driven fluidic medium flow reducing the cost of the system dramatically since the purchase of a pump system was not necessary (Lei et al., 2014). The study concluded that this model is more accurate than other current *in vitro* models and contains the potential to eventually lead to personalized medicine as a result of the utilization of patient-derived cells (Lei et al., 2014).

Although organ chip models have the potential to be useful tools to screen anticancer drug therapies, they require careful planning and precise execution. Organ chip models are still far from perfect and have improvements to be made. A challenge that exists in them today include them being more difficult to use than other 3D culture techniques (Sontheimer-Phelps et al., 2019). Another challenge has to do with how fragile some models can be; the complexity of the microsystems can cause experiments to be interrupted by something as small as the formation of a single bubble in the chamber (Sontheimer-Phelps et al., 2019). Lastly, cell structural integrity and functionality are often times limited in long-term experiments when using common media (Sontheimer-Phelps et al., 2019). As organ chip models improve and more accurate replicable organ models arise, there will be less of a need for animal models allowing for cheaper and more environmentally friendly drug screening processes.

## Tumor Models and Immunotherapy

Understanding tumor characteristics by developing an accurate tumor model is the key to understanding the link between today’s various types of cancers. 3D tumor cells grown using 3D cell culture methods have claimed the spotlight in tumor cell biology research because of their innate ability to replicate the *in vivo* environment of a tumor cell *in vitro*. Although 2D cell culture techniques are still commonly used because of their convenience, their inability to mimic the pathophysiology of tumor cells often renders their use impractical due to their inaccurate response to radiation therapy and drugs (Dunne et al., 2014). Cancer cell aggregates known as MCTS are grown using 3D culture methods via suspension or embedment in gels (Lv et al., 2017). MCTS grown using these methods allow for models that mimic the *in vivo* tumor microenvironments (Xu et al., 2013; Imamura et al., 2015; Lv et al., 2017). MCTS can be grown via static suspension, hanging drop methods, magnetic levitation, spinner bioreactor, rotational bioreactor, microfluidic system, and gel embedding (Lv et al., 2017; 20). These various methods allow for the replication of different microenvironments that can be found in specific types of tumors.

Tumor models have also been used to study cellular signaling pathways in which cellular pathways can be mapped and compared to cells in a 2D cell culture model to determine if 2D models are viable or not. If the 2D model is unrepresentative of the 3D *in vivo*-like model, then researchers can assume the cellular signaling pathways in the 2D are inaccurate (Lovitt et al., 2014). Furthermore, 3D cancer models have also been used extensively in the study of gene expression. One study compared a 2D monolayer cell culture to a 3D cell culture in which 24 malignant and non-malignant breast cell lines were cultured generated by lrECM (Barcellos-Hoff et al., 1989; Petersen et al., 1992). Big discrepancies in gene expression were uncovered for genes encoding signal transduction proteins, leading researchers to conclude that cellular pathways vary between 2D and 3D cultures established on lrECM (Li et al., 1987). Gene expression alterations were also discovered for malignant and non-malignant prostate cell lines when a similar test was conducted.

Much like organ-on-a-chip models, tumor-on-a-chip models have gained increasing popularity for the same reason as organ chips. A glioblastoma tumor was grown on a chip using C6 cells and treated with magnetic hyperthermia therapy (Yu et al., 2019). After the cells were seeded in the 3D culture, magnetic nanoparticles were injected into the central cavity of the chip allowing them to come into contact with the 3D cell culture thus submitting them to an alternating magnetic field (Yu et al., 2019). A fluorescence assay was used to assess the efficacy of the magnetic hyperthermia treatment (Yu et al., 2019). The study concluded that all the tumor cells on the chip were lysed after 30 min of treatment (Yu et al., 2019). Although the study contained a limiting factor due to the lack of vascular network typically present in the tumor tissue, the study proved that organ chip methods of drug testing in glioblastomas hold high potential in future studies (Yu et al., 2019).

A scrupulous investigation was conducted that aimed to mimic the progression of kidney cancer via a novel 3D metastatic cancer cell model (Wang et al., 2020). Previous metastatic cancer models have been created culturing cells in 3D but lacked the ability to interact with the correct physiopathological conditions as well as accurately reflect the effect of anticancer drugs *in vivo* (Wang et al., 2020). A 3D biomimetic liver microtissue modeled in DLM/GelMA hydrogel and subjected to continuous perfusion was used to culture the kidney cancer cells (Caki-1) (Wang et al., 2020). This served as an effective model to mimic kidney cancer metastasis and discovered a linear anti-cancer correlation between the concentration of Caki-1 cells and the concentration of the drug 5-Fluorouracil.

Immunotherapy is an area of research that has quickly gained popularity. Immunotherapy methods use the patients’ own immune system and either enhance the natural response to tumor antigens, or direct specific attacks on malignant cells (Sherman et al., 2018). Typically, when 2D tumor cultures are treated with immunotherapy treatments, attrition rates are high due to the 2D model’s inability to replicate the three-dimensional complex characteristics of a tumor (Sherman et al., 2018). In contrast, 3D cell cultures provide researchers with the ability to replicate the tumor model by successfully mimicking the 3D cell-matrix formation (Dangles-Marie et al., 2003; Sherman et al., 2018). This allows for the immune cells to attack the malignant target cells, much like an *in vivo* model would (Holmes et al., 2011). Studies have shown that 3D-cultured tumor cells have greater resistance to cytotoxicity as a result of phenotypic changes that are not present in 2D-cultured tumor cells (Edmondson et al., 2014). A study conducted by Dangles-Marie et al. found that in 3D culture of a lung carcinoma cell line, there was a decrease in Hsp70, and thusly a decrease in antigen presentation (Sherman et al., 2018). This decrease in antigen presentation caused cytotoxic T lymphocyte attacks to become less likely in the cells, making it evident that 3D tumor models resemble the *in vivo* tumor microenvironment more accurately than 2D tumor models (Holmes et al., 2011).

Due to recent success in treating melanoma skin cancer, researchers have begun modeling melanoma tumor cells in 3D culture spheroids to target molecular mechanisms aiding in resistance in current immunotherapy treatments (Müller and Kulms, 2018). Melanoma spheroids are grown *in vitro* to model *in vivo* tumor cells by using juvenile primary fibroblasts and keratinocytes (Müller and Kulms, 2018). Juvenile primary fibroblasts and keratinocytes can either be isolated from a sample of juvenile foreskin or can be purchased and are advantageous because they usually are less differentiated than adult primary skin cells (Müller and Kulms, 2018). 3D melanoma spheroids are generated via the hanging drop method and provide researchers with the ability to study drug resistance within the cells (Lin and Chang, 2008). By using these methods, scientists are able to create tumor models on a patient-to-patient basis to test treatments, as well as develop general tumor models to test a variety of non-patient specific treatments (Lin and Chang, 2008).

In an effort to understand how primary lung cancer progresses to metastatic lung cancer, one study used 3D cell culturing techniques to plot the migration of the cancer cells. Matrigel invasion assays were implemented on serum-starved cells in which non-invaded cells were removed after 24 h, and the chambers were stained with crystal violet to view the invaded cells (Xiong et al., 2019). 3D invasion assays were also performed on HN12 cells and seeded in the SeedEZ 3D ring (Xiong et al., 2019). The cells were stained with Texas-red phalloidin after 10 days of growth and viewed under a fluoresce microscope (Xiong et al., 2019). What the study uncovered through the use of these methods, is that NAP1/NCKAP1 is highly correlated with primary NSCLC and metastasis relative to normal lung tissues (Xiong et al., 2019). Furthermore, the overexpression of NAP1 causes MMP9 activation thus invoking invasion and metastasis (Xiong et al., 2019). The usage of Matrigel and 3D invasion assays were crucial to understanding the link between NAP1 and MMP9 in order to help understand how primary lung cancer progresses to metastatic lung cancer.

A novel 3D bone marrow niche model was assembled to study the effects of a new class of engineered immune cells known as TEGs (αβT cells engineered to express a defined γδTCR) on primary myeloma cells (Braham et al., 2018). TEGs proved their ability to migrate through the 3D culture as well as initiate a killing response directed at the primary myeloma cells (Braham et al., 2018). Prior to this experiment, no 2D models were effective in predicting the clinical success of a treatment highlighting the need for a patient-specific model supporting primary myeloma cells (Braham et al., 2018). Compared to the 2D models, the 3D model outperformed the 2D models with its ability to analyze specific homing as well as on- and off-target effects (Braham et al., 2018). With the help of 3D cell culture, this 3D bone marrow niche model allows studying novel immunotherapies, therapy resistance mechanisms, and possible side-effects of primary myeloma (Braham et al., 2018).

Lee et al. (2018) crafted a 3D microfluidic model used to assess the impact monocytes have on TCR T cells in the hepatitis B virus (HBV). Previous studies have confirmed that monocytes interrupt natural T cell functions, but little is known about the effects monocytes have on TCR T cells (Lee et al., 2018). To test the efficacy of the 3D microfluidic model, the 3D model was compared to standard 2D assays when testing the effect of monocytes on TCR T cells (Lee et al., 2018). What they found, was that retrovirally transduced TCR T cell cytotoxicity toward cancer cells was suppressed while mRNA electroporated TCR T cell cytotoxicity was unaffected in the presence of monocytes in the 3D microfluidic model (Lee et al., 2018). In the standard 2D assay, however, the monocytes did not suppress cytotoxicity toward cancer cells in either the retrovirally transduced TCR T cells or mRNA electroporated TCR T cells (Lee et al., 2018). These data suggest that the 3D microfluidic model provides a more accurate assessment when investigating tumor-immune cell behavior and has the potential to uncover the impact of specific biological pathways on monocyte-TCR T cell interactions (Lee et al., 2018).

## Tissue Engineering

When designing an *in vitro* model for a cell, it is imperative that the environment accurately represents a cell’s natural environment *in vivo*. One of the ways this is achieved is through proper TE techniques. TE was first introduced in 1988 at UCLA Symposia on Molecular and Cellular Biology by Professor Robert Nerem (Eltom et al., 2019). When designing a tissue, the most important aspect of the tissue model is properly mimicking the porosity of the tissue *in vivo*. Among the various methods that exist, the methods commonly used for scaffold production in 3D cell cultures are freeze-drying, SCPL, electrospinning (ES), and 3D printing (3DP). As TE methodologies become more advanced, it may become feasible to construct entire organs as well as repair damaged organs using patient cells to avoid rejection from the patient’s body (Zhu et al., 2016).

### Freeze-Drying

Freeze-drying is a process that is used to create highly porous PGLA scaffolds (Eltom et al., 2019). Through the homogenization of a polymer solution in an organic solution and water mixture, an emulsion is created (Liu and Ma, 2004). The emulsion is then rapidly cooled to keep it in a liquid state structure where freeze-drying is then implemented to remove the solvent and water ultimately maintaining the original 3D structure (Liu and Ma, 2004). Freeze-drying is an effective technique when making scaffolds with a porosity of more than 90% and a pore size anywhere from 20 to 200 μm (on average) (Liu and Ma, 2004).

Recent studies have shown that freeze-drying techniques used to create hydrogel scaffolds are effective in 3D cell culture. Zhou et al. (2019) created a novel ready-to-use scaffold for cell culture with a hybrid of gelatin and polypropylene non-woven fabric via freeze-drying. The scaffold’s structural integrity was unchanged after over 90 days in storage, making it a good candidate for 3D cell culture because of its read-to-use capability (Zhou et al., 2019). Furthermore, the addition of gelatin into the scaffold demonstrated an increase in porosity as well as liquid storage capability in 3D cell cultures (Zhou et al., 2019).

Bodenberger et al. (2017) took a novel approach to fabricate hydrogels from yeast whole cell protein via freeze-drying. Being that some hydrogels can be difficult to consistently replicate and are often expensive to order, yeast protein hydrogels present a cheap and potentially reliable alternative for hydrogel fabrication (Bodenberger et al., 2017). When freeze-drying yeast hydrogels, the pore size can be made as big as 100 μm and the hydrogels can absorb liquid up to 12 times their weight allowing cells to stay in a highly hydrated environment (Okay, 2009; Bodenberger et al., 2017). Being that pore size between 5 and 350 μm is sufficient for 3D cell culture and adequate diffusion rates were observed in the hydrogel, yeast hydrogels fabricated via freeze-drying show tremendous potential for 3D cell culture (Annabi et al., 2010; Bodenberger et al., 2017).

### Solvent-Casting Particulate Leaching

Solvent casting particulate leaching is a technique used to create porous scaffolds by mixing water-soluble salt particles into a biodegradable polymer solution and subsequently casting the mixture into the scaffold mold (Liu and Ma, 2004). The solvent is then removed via evaporation and the salt particles are leached out leaving behind a porous structure (Liu and Ma, 2004). SCPL is advantageous because it is relatively simple, and the pore sizes and porosity can be easily controlled by the size of the salt particles used and the salt/polymer ratio (Liu and Ma, 2004). The disadvantages, however, include limited interpore connectivity making uniform cell seeding and tissue growth difficult, residual salt particles being left over, and a thickness range of 0.5 to 2 mm for the scaffold as a result of soluble particles being difficult to remove from the interior of thick scaffolds (Liu and Ma, 2004).

Chia et al. (2019) developed an enhanced SCPL technique that involves an extra step of centrifugation to create 45S5 BG reinforced PU scaffolds (PU-BG). PU-BG scaffolds were created using different centrifugal speeds of 1500 rpm, 2000 rpm, 2500 rpm, and 3000 rpm (Chia et al., 2019). The porosity and integrity of the PU-BG scaffolds were then compared to those made using the conventional SCPL method (no centrifugation) (Chia et al., 2019). The scaffolds fabricated using the enhanced SCPL method contained a porosity of about 88% to 90% while the scaffolds created using the conventional SCPL method contained a porosity of about 81% (Chia et al., 2019). Furthermore, the scaffolds created via the enhanced SCPL method displayed high pore interconnectivity as a result of the centrifugation helping distribute the salt particles more evenly throughout the scaffold (Chia et al., 2019). As a result of centrifugation, however, the scaffolds created with the enhanced SCPL method displayed a lower compressive strength than the scaffolds made by conventional SCPL deeming them only capable for low load-bearing applications (Chia et al., 2019). Although scaffolds created via SCPL can be modified via the addition of a centrifugation step to have greater porosity and interconnectivity, the lack of compressive strength may pose difficulties when modeling bone tissue for bone tissue repair.

Sola et al. (2019) developed SCPL polymer scaffolds to mimic the bone marrow niche so that medical therapies can be tested on cancers such as lymphomas and leukemias *in vitro*. A flexible PU polymer and a rigid PMMA polymer were compared when using NaCl as the porogen (Sola et al., 2019). The PU scaffold had a porosity of 91% and a compression of 29kPa, while the PMMA scaffold had a porosity of 84% and a compression of 1283kPa (Sola et al., 2019). Upon a collagen-coating, it was observed that human stromal HS-5 cells stuck to the scaffold supports as well as retained their pro-survival action toward co-cultured cancer cells avoiding the drug’s cytotoxic effect (Sola et al., 2019). These scaffolds are effective for mimicking the bone marrow microenvironment and have the potential to be extremely effective in preclinical drug studies.

### 3DP Scaffolds for 3D Cell Culture via Electrospinning

3D printing has also allowed the printing of complex ECM-like scaffolds with such control that details can be fine-tuned at the micrometer level (Do et al., 2015). A common method of printing scaffolds is a method known as electrospinning (ES). Electrospinning utilizes an electric field as a control mechanism to form and deposit polymer fibers onto a specific substrate (Liu and Ma, 2004). A charge imbalance is created upon the injection of an electrical potential into a melt or polymer solution (Reverchon et al., 2012). The Taylor cone is a stream of liquid produced at the critical point when the polymer solution is exposed to a high voltage causing it to become charged (Reverchon et al., 2012). The fibers are formed as the solvent evaporates off during the travel of the stream to the target (Xue et al., 2019). ES is among the most established TE techniques as electrospun collagen is commonly used to create tissue scaffolds due to how well it mimics the natural ECM (Ma, 2004).

k When collagen is made, it is typically made with highly toxic organic solvents such as HFIP (Türker et al., 2019). Türker et al. (2019) designed a new way to create collagen scaffolds without the use of toxic organic solvents by using the sacrificing agent PVP instead of HFIP. By using the biodegradable synthetic polymer PLLCL as a base scaffold for the integration of collagen type I (Col), biomimetic PLLCL/PVP/Col hybrid scaffolds were created using co-electrospinning techniques (Türker et al., 2019). After ES was complete, the PVP was removed from the scaffold by being solubilized in water (Türker et al., 2019). To test whether the hybrid scaffold could support a 3D cell culture, NIH 3T3 mouse fibroblast cell line was cultured (Türker et al., 2019). The cells were successfully grown over a 14-day span and the results indicated that the hybrid PLLCL/Col scaffold promotes cellular adhesion and proliferation even during long-term studies much more effectively than standard 2D cell culture models (Türker et al., 2019).

Permlid et al. (2019) demonstrated a novel animal-friendly 3D electrospun polycaprolaceton (PCL) synthetic scaffold that effectively mimicked the collagen network of tissue. Human breast cancer cell lines JIMT-1 and MCF-7, the normal-like breast epithelial MCF-10A cell line, and mouse L929 fibroblasts were seeded in the 3D PCL scaffold and incubated for 7 days (Permlid et al., 2019). After 7 days, the 3D cultures were analyzed using scanning electron microscopy, confocal laser scanning microscopy, and cryosectioning (Permlid et al., 2019). The results showed that both the malignant as well as normal cell lines flourished in the 3D PCL scaffold indicating the potential to create a tumor ex vivo platform to screen therapeutic compounds (Permlid et al., 2019).

Sankar et al. (2019) used ES in conjunction with photolithography to fabricate both nano- and micro-patterned PLGA/Collagen/nHAp fiber mats. 2D scaffolds and 3D scaffolds were used with MSCs to study the effect of geometric cues on proliferation and differentiation of MSCs (Sankar et al., 2019). The MSC were seeded on the 2D scaffolds while MSC spheroids that were cultured for 3 days prior to seeding were seeded on the 3D scaffolds (Sankar et al., 2019). The results indicated that higher osteogenic differentiation was found in the 3D spheroids than the 2D cells (Sankar et al., 2019). This strategy of seeding 3D spheroids along with patterned substrates that resemble the natural tissue architecture may hold the power to help in regenerating a functional bone tissue (Sankar et al., 2019).

### 3DP Scaffolds for 3D Cell Culture via Stereolithography

Stereolithography is another common method used to create artificial scaffolds. Stereolithography is a methodology of 3DP that prints an UV curable material in thin sheets layer-by-layer until the scaffold is complete (Eltom et al., 2019). Each layer is laid on top of one another following the drying of the subsequent layer (Eltom et al., 2019). After the scaffold is finished printing, it is placed under a UV light where it is postcured (Eltom et al., 2019).

Creff et al. (2019) fabricated a 3D model of the intestinal epithelium *in vitro* by combining a photopolymerizable hydrogel that promotes the growth of intestinal cell lines with stereolithography 3DP. Caco-2 intestinal epithelial cells were grown on the scaffold for 2 weeks and showed much higher rates of differentiation than standard 2D cultures (Creff et al., 2019). Thus, this model is a great candidate for studying intestinal homeostasis and regeneration mechanisms *in vitro* (Creff et al., 2019).

Lee S.J. et al. (2017) combined stereolithography with electrospinning to create an advanced neural network scaffold. The inclusion of electrospinning fibers into the scaffold indicated significant improvements in neural stem adhesion when compared to 3D models without the added fibers (Lee S.J. et al., 2017). There were two types of fibers added to the 3D models: PCL fibers and PCL/gelatin fibers (Lee S.J. et al., 2017). What they discovered was the PCL/gelatin fibers enhanced the neural stem cell differentiation when compared to the PCL fibers without gelatin (Lee S.J. et al., 2017). The results of this study indicate that there is a high potential for developing unique 3D neural tissue models by implementing electrospinning and stereolithography techniques (Lee S.J. et al., 2017).

### 3DP Microfluidic Devices for 3D Cell Culture

3D printing has allowed researchers to design microfluidic devices on the computer and then print them out using a 3D printer (Eltom et al., 2019). Microfluidic devices are commonly made with polydimethylsiloxane but due to inconsistent reproducibility from lab-to-lab, some people have begun using 3DP to make them (Castiaux et al., 2019a). Physiologically relevant dimensions can be reached within the channels of microfluidic devices via extrusion-based printing, stereolithography, and PolyJet (Waheed et al., 2016). Castiaux et al. (2019a) demonstrate two novel techniques to 3DP enclosed microfluidic channels via a PolyJet 3D printer. The first way implements a liquid to support cover layer prints while the second method uses a polycarbonate membrane to support the additional layers (Castiaux et al., 2019b).

Shimizu et al. (2019) designed an ECM collagen-based stretchable microfluidic system that resembles the *in vivo* blood vessels and allows for *in vitro* 3D cell culture. The ECM microfluidic channel was created using 3D printed water-soluble sacrificial molds (Shimizu et al., 2019). The stretchable design mimics the *in vivo* environment by allowing cells to be cultured in 3D while fluid shear stress and mechanical stretching occur simultaneously (Shimizu et al., 2019). This model could potentially be useful for studying vascular tissue formation due to its simple design and replicability.

## Challenges and Future Perspective

3D cell culturing methods stand at the precipice of groundbreaking discovery and have the potential to unlock the answers researchers have been unable to uncover through the use of 2D cell culture techniques. With new technology however, comes obstacles and challenges. Although advantageous in many ways over 2D cultures, 3D culture tends to be more expensive and can be difficult to replicate cell microenvironments when using certain 3D culture methods (Langhans, 2018). Furthermore, matrices often have multiple components that make them difficult to construct and require extensive amounts of labor (Antoni et al., 2015).

Imaging also becomes difficult when large scaffolds are used because there is a limit when scaling a single 3D format (Antoni et al., 2015). Anchorage-dependent cultures such as hanging-drop plates and ultra-low-attachment plates can also be very difficult to image due to plate incompatibility with microscopes and uncentered spheroids in well plates (Booij et al., 2019). The most common way to analyze cellular phenotypes is by using conventional wide-field or confocal fluorescence microscopy (Booij et al., 2019). Fluorescence microscopy is often still challenging in 3D cell cultures because unlike 2D cell culture where only a single *xy* image is taken, 3D cell cultures must obtain a *z* stack by taking a series of *xy* images at fixed intervals in the vertical direction by automated microscopes (Booij et al., 2019). Having to take a series of *xy* images to obtain a *z* stack often increases the time significantly and as a result, higher magnification objectives (40–60×) are currently not practical for high-throughput setting as it takes too much time and storage space (Booij et al., 2019).

Flow cytometry is a common technique used for 2D cell cultures to count cells, detect microorganisms, sort cells, detect biomarkers, detect protein engineering, and determine cell characteristics and functions (Picot et al., 2012). Flow cytometry has been used on 3D spheroids but requires the dissociation of the spheroids into a single-cell suspension via an enzyme such as trypsin and mechanical disruption (Gong et al., 2019). Because the spheroids must be broken up into a single-cell suspension, it ultimately becomes an endpoint assay as the cells are disposed following the completion of flow cytometry (Gong et al., 2019).

Another common issue facing 3D cell culturing techniques is the automation of liquid handling (Booij et al., 2019). Liquid handling for suspension media and ultra-low-attachment can be easily automated, but more viscous liquids such as collagen- and Matrigel-containing hydrogels present unique challenges (Booij et al., 2019). Temperature sensitive polymerization in these gels requires quick liquid handling and careful environment control to avoid premature polymerization (Lei et al., 2014). Automation can often be achieved for many 3D culturing techniques in 96- or 384-well plates but further automation in miniaturized models may prove difficult as pipetting volumes are so small (Lei et al., 2014).

A novel benchtop bioreactor was recently designed and tested in an article by de Bournonville et al. (2019). The bioreactor was built to allow the user to control the internal environment as well as use it in a small space outside of clean room environments. The bioreactor proved to be effective in supporting scaffold-based 3D progenitor cell cultures as well as presented the ability to provide solutions for automated cell therapy bioprocessing.

Despite the limitations currently facing 3D cell culture methods, a survey from the HTS technologies found that two-thirds of people surveyed have plans to switch from 2D cell culture to 3D cell culture, with many of them having already switched (Antoni et al., 2015). The more researchers who switch over to 3D, the quicker new methodologies will be developed that overcome the current limitations facing 3D cell culture. Likewise, many scientists already have plans for the future of 3D cell culture. The future of organoids remains bright, with the potential for developing alternative organ transplantation procedures as well as tumor models via patient-derived polypotent stem cells (Cushing and Anseth, 2007). Immunotherapy in 3D cell culture models is one of the most hopeful methods due to recent success relating to cancer treatment (Sherman et al., 2018). As 3D tumor models become more advanced, immunotherapy treatments will advance to the point where more clinical trials can be performed with the potential of eventually finding a treatment for various cancers.

## Conclusion

Both 2D and 3D cell culture techniques provide methods which are necessary for advancing research. 3D cell culture, however, has proven it has the potential to completely change the way in which new drug treatments are tested, diseases are modeled, stem cells are utilized, and organs are transplanted. As 3D cell culture becomes more commonplace, the techniques will be better understood, and more advanced methods will arise. Researchers currently working to test new drug therapies via 2D cell culture models should seriously consider 3D cell culturing options. The benefits of co-culturing cells in 3D are superior to that of 2D cell culturing and as the techniques for tissue engineering improve, tumor models, cancer treatment therapies, and disease testing methodologies will improve.

## Author Contributions

YT conceived the original idea. CJ wrote the manuscript with supervising from YT. Both authors provided critical feedback and helped to shape the manuscript.

## Conflict of Interest

The authors declare that the research was conducted in the absence of any commercial or financial relationships that could be construed as a potential conflict of interest.

## Abbreviations

2D: two-dimensional
3D: three-dimensional
3DP: 3D printing
b/w: black and white
BBB: blood brain barrier
BBBC: blood brain barrier chip
BG: bioactive glass
Col: collagen type I
ECM: extracellular matrix
ES: electrospinning
GFP: green fluorescent protein
GI: gastrointestinal
HFIP: hexafluoro isopropanol
hIEC: human intestinal epithelial cells
lrECM: laminin-rich-extracellular matrix
MCTS: multicellular tumor spheroids
MCTS: multicellular tumor spheroids
MMP9: Matrix metalloproteinase 9
MOC: multiple-organs-on-a-chip
MSC: mesenchymal stem cells
NAP1/NCKAP1: Nck-associated protein 1
nHAp: nanohydroxy apatite
NSCLC: non-small cell lung cancer
PGLA: poly(lactic-co-glycolic acid)
PLLCL: poly(L-lactide-co–caprolactone
PMMA: polymethyl methacrylate
PU: polyurethane
PVP: polyvinylpyrrolidone
SCPL: solvent-casting particulate leaching
TCR T cells: T cell receptor-redirected T cells
TE: tissue engineering
UV: ultraviolet.
